# UVR-sensor wearable device intervention to improve sun behaviors and reduce sunburns in melanoma survivors: study protocol of a parallel-group randomized controlled trial

**DOI:** 10.1186/s13063-020-04881-3

**Published:** 2020-11-23

**Authors:** Rachel I. Vogel, Rebekah H. Nagler, Rehana L. Ahmed, Katherine Brown, Xianghua Luo, Brian C. Martinson, DeAnn Lazovich

**Affiliations:** 1 Department of Obstetrics, Gynecology and Women’s Health, Division of Gynecologic Oncology, University of Minnesota, Minneapolis, MN USA; 2 Masonic Cancer Center, University of Minnesota, Minneapolis, MN USA; 3 Hubbard School of Journalism & Mass Communication, University of Minnesota, Minneapolis, MN USA; 4 Department of Dermatology, University of Minnesota, Minneapolis, MN USA; 5 Division of Biostatistics, School of Public Health, University of Minnesota, Minneapolis, MN USA; 6 HealthPartners Institute, Bloomington, MN USA; 7 Center for Care Delivery and Outcomes Research, Minneapolis Veterans Affairs Health System, Minneapolis, MN USA; 8 Department of Medicine, University of Minnesota, Minneapolis, MN USA; 9 Division of Epidemiology and Community Health, School of Public Health, University of Minnesota, Minneapolis, MN USA

**Keywords:** Melanoma, Sun protection, Ultraviolet exposure, Cancer survivors, Ecological momentary intervention

## Abstract

**Background:**

Individuals who have been diagnosed with melanoma have more than a 9-fold increased risk of developing another melanoma. Ultraviolet radiation (UVR) exposure following a melanoma diagnosis can be modified to reduce risk of a new melanoma diagnosis. Yet research shows that many melanoma survivors do not report optimal sun protection practices. The objective of this study is to evaluate the effectiveness of a UVR-sensor wearable device to improve sun protection behaviors and reduce sunburns in a randomized controlled trial (RCT) in melanoma survivors.

**Methods:**

We will conduct an RCT among 368 melanoma survivors in two waves (Summer 2020, Summer 2021). This approach allows for adequate recruitment of the required sample and potential improvements to recruitment, compliance, and retention strategies between waves. The intervention includes an informational brochure about sun protection behaviors and a commercially available UVR-sensor wearable device (Shade), which accurately measures UVR. The device, along with its associated mobile application, measures and stores UVR exposure. As UVR exposure accumulates, the device provides notifications to increase sun protection action. Survivors in the control group receive the device and a separate mobile application that does not provide notifications or summary UVR exposure data. Participants will be asked to wear the device for 12 weeks. They will complete surveys about their sun behaviors at study entry, every 4 weeks during the intervention, and 1 year later. At the end of the intervention period, intervention and control groups will be compared for differences in a summary measure of sun protection habits and experience of a sunburn. We will also measure self-reported physical activity, depression, and anxiety to examine potential unintended negative consequences of the intervention.

**Discussion:**

The study intervention will be completed Fall 2021, with anticipated results available in 2022. If this intervention improves sun protection behaviors in melanoma survivors, these findings would support expanding the use of this technology with other populations at high risk for melanoma.

**Trial registration:**

ClinicalTrials.gov NCT03927742. Registered on April 15, 2019.

## Administrative information

The order of the items has been modified to group similar items (see http://www.equator-network.org/reporting-guidelines/spirit-2013-statement-defining-standard-protocol-items-for-clinical-trials/).

**Table:** 

Title {1}	UVR-sensor wearable device intervention to improve sun behaviors and reduce sunburns in melanoma survivors: study protocol of a parallel-group randomized controlled trial
Trial registration {2a and 2b}.	ClinicalTrials.gov, #NCT03927742. All items on the WHO Trial Registration Data set are included within this manuscript and available on ClinicalTrials.gov.
Protocol version {3}	05/08/2020, Version 3.1
Funding {4}	This study is funded by the Melanoma Research Alliance: The Wayne Stinchcomb Big Orange Foundation – MRA Young Investigator Award (568166) and the American Cancer Society Research Scholar Award (133512-RSG-19-014-01-CPPB). Preliminary studies for this protocol were supported by the Masonic Cancer Center of the University of Minnesota grant NIH P30 CA77598. Use of data collection and management tools are supported by the National Institutes of Health’s National Center for Advancing Translational Sciences, grant UL1TR002494.
Author details {5a}	University of Minnesota, Minneapolis, Minnesota, USA HealthPartners Institute, Bloomington, Minnesota, USA
Name and contact information for the trial sponsor {5b}	PI: Rachel I. Vogel, University of Minnesota, 420 Delaware Street SE MMC 395, Minneapolis, MN, USA, email: isak0023@umn.edu.
Role of sponsor {5c}	The funders do not have any role in study design, data collection and management, writing of reports or decision to submit reports for publication.

## Introduction

### Background and rationale {6a}

Melanoma, one of the most serious types of skin cancer, has been increasing in incidence over the past 30 years. With a 5-year survival rate of 93%, there are currently over one million melanoma survivors in the USA [1]. Melanoma is considered a generally preventable cancer, with excessive ultraviolet radiation (UVR) exposure being one of the strongest risk factors for the disease [2, 3]. Patients diagnosed with melanoma experience high rates of recurrence and second melanomas, with an approximately 9-fold increased risk of developing another melanoma [4]. Patients who recur or have a second melanoma diagnosis have a significantly worse prognosis [5, 6]. Importantly, UVR exposure following a melanoma diagnosis can be modified to reduce risk of a new melanoma diagnosis [7]. Our recent study found that a significant subgroup of melanoma survivors (20%) experienced sunburn in the past year, reported intentional tanning (10%), did not wear a hat (67%) or stay in the shade (52%), and spent more than 2 h in the sun on weekends between 10 am and 4 pm (75%), putting them at elevated risk for future melanomas. Therefore, while some melanoma survivors are reporting healthy UVR exposure and protection behaviors, opportunities remain to reduce future melanoma risk in the majority of melanoma survivors.

The use of wearable technology devices has grown quickly over the last decade. The techniques implemented in these devices to promote behavior change vary widely, from self-monitoring and goal setting to social comparison and prompts/cues [8]. These devices often monitor physical activity versus sedentary behavior and interface with a computer/mobile phone application to provide feedback and promote goal setting. Studies using wearable technology devices to promote physical activity and weight loss have been promising, as they provide frequent and current information and triggers to action [9]. Studies in cancer survivors to improve physical activity have found these devices to be acceptable and usable, with the large majority reporting confidence in operating the basic features of the devices and wearing the device about 80% of the time [10–13]. Newer devices have UVR sensor/monitoring systems and associated behavior prompts, though they have not yet been studied to promote behavior change for sun exposure and protection.

This study will focus on testing a wearable technology intervention to increase sun protection behaviors and reduce sun exposure and sunburns among melanoma survivors. This intervention will serve as a first step toward the long-term goal of reducing second primary melanoma diagnoses among melanoma survivors.

### Objectives {7}

The objective of this trial is to test the effectiveness of a wearable device with UVR-sensing technology (Shade, version 2) and corresponding mobile application intervention to promote healthy sun behaviors in melanoma survivors. We hypothesize that participants randomized to the intervention group, compared to those assigned to the control group, will report greater sun protection and avoidance practices and fewer sunburns during the intervention period.

### Trial design {8}

We will conduct a parallel randomized, controlled, superiority trial (RCT). Participants will be randomly assigned 1:1 to either the intervention or the control group for 12 weeks and surveyed regarding their sun protection and surveillance behaviors at study entry, every 4 weeks during the intervention, and 1 year later. Randomization is stratified by the factors age, gender, year of diagnosis, and cancer stage using a block randomization scheme with random blocks to improve balance. The RCT will be conducted among 368 melanoma survivors in two waves (Summer 2020, 2021) in Minnesota. This two-wave summer method has been utilized successfully by Glanz et al. [14] and will account for the seasonality of UVR exposure, allow for adequate recruitment of the required sample size, and allow for potential improvement in methods for recruitment, compliance, and retention across waves.

## Methods: participants, interventions, and outcomes

### Study setting {9}

We will recruit 368 individuals with a history of invasive cutaneous melanoma in the USA from HealthPartners health care system, based in Minnesota. HealthPartners is a large consumer-governed nonprofit health care organization, providing care, coverage, research, and education to improve health and well-being in partnership with its members, patients, and community. All study interactions will happen remotely via phone, mail, and email.

### Eligibility criteria {10}

Eligible participants will be 18–75 years of age, previously diagnosed with stage I–IV cutaneous invasive melanoma, able to read/write in English, smartphone owners, and able to provide voluntary informed consent. Participants will be excluded if they participated in early phases of the study, if they had stage 0 (in situ) melanoma, including lentigo maligna, or other melanoma such as uveal or mucosal, are unable to provide informed consent, are currently pregnant, or have opted out of their records being used for research purposes.

### Who will take informed consent? {26a}

A phone interviewer with HealthPartners will describe the study and obtain verbal consent from potential trial participants and contact information (name, address, phone number) to be shared with members of the University of Minnesota research staff. The University of Minnesota staff will then send an introductory email that includes a link to complete an online consent form. Online consents and Health Insurance Portability and Accountability Act (HIPAA) forms will obtain signatures captured electronically via the e-consent functionality in Research Electronic Data Capture (REDCap) [15]. Potential participants are encouraged to reach out to the study team at any point with questions or concerns prior to and after providing consent.

### Additional consent provisions for collection and use of participant data and biological specimens {26b}

All participants will complete online HIPAA authorization forms, enabling the study team to seek data from HealthPartners and store information regarding participants’ melanoma diagnosis, including date of diagnosis and disease stage, along with self-reported health data.

## Interventions

### Explanation for the choice of comparators {6b}

The control group will also be given the UVR-sensor-enabled device to wear (details below); however, they will be provided a version of the mobile app that only collects the UVR exposure and does not provide notifications as they reach daily limits or summary information about exposure. The attention-control group was chosen over no intervention for three reasons: one, we can objectively measure UVR exposure in both groups; two, providing the device to the control group will help with recruitment, engagement, and retention; and three, the intervention is low risk.

### Intervention description {11a}

We are adopting the Health Belief Model as our theoretical guide for this intervention given its emphasis on several constructs important for promoting behavior change, including individual perceptions—such as perceived susceptibility, perceived severity, perceived benefits, perceived barriers, and self-efficacy—and cues to action, or factors in the environment that can trigger action [16, 17]. Distinct intervention components, described below, will enable us to address individual perceptions and provide cues to action to prompt engagement in optimal sun protection behaviors and reduce sunburns.

We will provide a commercially available UVR-sensor-enabled wrist wearable device to all participants, Shade, 2nd generation, which was developed in collaboration with the National Health Institutes (National Cancer Institute). Shade reported significantly more accurate UVR measurements than similar devices [18], and melanoma survivors have previously reported that the first version of the device was easy to use [19]. In addition to accurately measuring UVR exposure in a manner that does not rely on self-report and memory, Shade includes a mobile app that helps users set daily limits that take into account UVR intensity, time of exposure, and sunscreen application. Importantly, the app and device provide prompts when approaching the daily limit, with the goal of serving as a cue to action. The device will alert participants once they reach 20% of their daily limit (and every 20% after that) via a notification on the participant’s phone. Participants are asked to wear the devices and sync it with the mobile app every day for 12 weeks, regardless of planned outdoor activities.

For participants in the intervention group, we also developed a brochure that provides information about the importance of UVR avoidance and protection among individuals with a history of melanoma and tips for making these behaviors routine and sustainable, with the goal of addressing perceptions to promote sun protection behaviors. The brochure will be provided to those in the intervention group as a printed brochure at baseline and via a website after completing each subsequent survey. The brochure was pretested among five individuals with a history of melanoma.

### Criteria for discontinuing or modifying allocated interventions {11b}

Participants will not be terminated from this study unless they request it. They will not be removed due to new melanoma diagnosed or recurrence unless requested. All participants, regardless of their device use, will be followed unless they drop out of the study. Switching between the intervention and control groups is not permitted. Those in the intervention group are not able to alter or turn off receipt of UV exposure notifications.

### Strategies to improve adherence to interventions {11c}

The study coordinator will view the data of all participants (both intervention and control) consistently to monitor device use and will contact participants who do not use their device or sync the app data more than 3 days in any given week to determine barriers and encourage use. In addition, a lottery-based incentive will be used to promote adherence. This incentive structure will indirectly support behavior change by incentivizing the use of the devices rather than directly incentivizing sun protection behavior. Participants will be entered into weekly lotteries for $100 gift cards for the duration of the intervention period. The weekly winners will be randomly drawn from all participants. The randomly selected winners will only be eligible for the prize if they wore the device at least 5 days during that week. We will email the study group every week indicating that the lottery was drawn, a winner was chosen, and the number of draws required to obtain a participant who was eligible to win.

### Relevant concomitant care permitted or prohibited during the trial {11d}

Participation in this study will not affect clinical care.

### Provisions for post-trial care {30}

There are no processes in place to provide post-trial care due to the minimal risks associated with this study and the return of the devices following the intervention period.

### Outcomes {12}

The primary outcome is a composite measure of healthy sun protection behaviors at the end of the intervention period (12-week survey) [14]. A total score for each individual will be created by taking the average of 6 protective behaviors (wearing a shirt with sleeves, wearing sunglasses, staying in the shade, using sunscreen, limiting time in the sun, and wearing a hat) on a 4-point ordinal scale ranging from 1 = rarely or never to 4 = always. This composite measure has been used previously in similar intervention studies [14]. We will not focus on change from baseline, as the baseline may be obscured by the time of year; further, we expect the baseline measures would be balanced between the two randomized groups, but we will take any differences into account in the analysis. As a self-reported outcome, social desirability may affect reported measures. It is possible that we will have misclassification of these measures; however, we do not anticipate misclassification rates to be differential by treatment group. While assessing the maintenance of behavior change is beyond the scope of the proposed study, we have added a short follow-up survey 1 year post study completion (64 weeks: the end of each following summer) to obtain preliminary estimates of durability.

A key secondary outcome for this study is a self-reported measure of at least one red or painful sunburn in the past 3 months at the completion of the intervention period (12-week survey) [20]. The National Institutes of Health has deemed sunburns following an intervention to be a clinically relevant endpoint and suitable outcome for trials aiming to prevent skin cancer [21]. We will calculate the proportions of individuals with at least one sunburn. We will additionally calculate the proportions of participants engaging in each sun protection behavior separately used in the calculation of the primary outcome and the number of hours outside per day between 10 am and 4 pm in the summer separately for weekdays and weekend days (week 12). For the purposes of this analysis and ease of interpretation, all measures were dichotomized into “optimal” and “suboptimal” categories. The following will be considered optimal: weekday sun exposure in the summer of 1 hour or less, weekend day sun exposure in the summer of 1 hour or less, and reporting often or always using sunscreen, often or always wearing a shirt with sleeves, often or always wearing a hat, often or always staying in the shade, and rarely or never intentionally tanning. The Shade device will provide objective measures of daily UVR exposure (continuous) and will be summarized as a total accumulation during the study period. Lastly, we will calculate a total sun protection knowledge score, which will be measured using six items created by the study team which address particular concerns related to sun protection following melanoma. We will create a total score by summing the number of correct answers (range 0 to 6), focusing on both the week 12 measure and change from baseline.

We will also measure physical activity, depression, and anxiety to examine any potential unintended negative consequences of the intervention. A number of potential harms of promoting sun protection have been suggested in the literature, including reduced physical activity and increased anxiety. A study of prospective cohorts found increased leisure-time physical activity was associated with risk of melanoma [22]. This finding is in accordance with recent cross-sectional data suggesting sunburn was greater among those who were highly physically active and that physical activity frequently occurs outdoors [23]. Increasing anxiety is another concern; one study found a higher proportion of participants in the intervention group reported greater rates of worrying about developing melanoma compared to control participants, although this difference was not statistically significant [24]. Conversely, it is possible that promoting sun protection behaviors may, in fact, improve emotional health by educating survivors about how to protect themselves when they are outside. Some survivors, at least initially, attempt to completely avoid the sun or be outside due to fear [25], and therefore empowering them to safely enjoy their activities may result in lower anxiety and depression. Physical activity will be measured using the Godin Leisure-Time Exercise Questionnaire [26] which asks about times per week of engaging in strenuous (vigorous), moderate, and mild physical activity on average of the preceding month, as well as minutes per week engaging in each type. We will calculate a total weekly leisure activity score: (9 × strenuous) + (5 × moderate) + (3 × light) and focus on total scores at week 12 and change from baseline. Depression and anxiety will be measured using the Hospital Anxiety and Depression Scale (HADS) instrument which includes 14 items designed to screen for potentially clinically relevant anxiety and depression [27]. Each item is scored from 0 to 3. We will calculate subscale anxiety and depression scale scores and the proportions of individuals with potentially clinically meaningful depression and anxiety using an established cut-off of 8 for each scale. We will focus on proportions with potentially clinically meaningful depression and/or anxiety at week 12 and changes in subscale scores between week 12 and baseline.

We will measure satisfaction and usability of the device at week 12 using the System Usability Scale [28]. The scale includes 10 items with Likert scale responses (strongly agree to strongly disagree). Total scores will be created by summing items and multiplying by 2.5 to obtain a range of 0–100.

Last, we will examine a number of metrics regarding study implementation, including recruitment, compliance/engagement, and retention. The implementation metrics will be examined after Wave 1 (Summer 2020) is completed to identify areas of opportunity to improve recruitment, compliance/engagement and retention that can be implemented prior to starting Wave 2 (Summer 2021). While we will keep the intervention consistent across waves, we will consider alternative methods of recruitment, incentives, reminders, and other actions to maximize engagement and retention.

### Participant timeline {13}

**Fig. 1:**
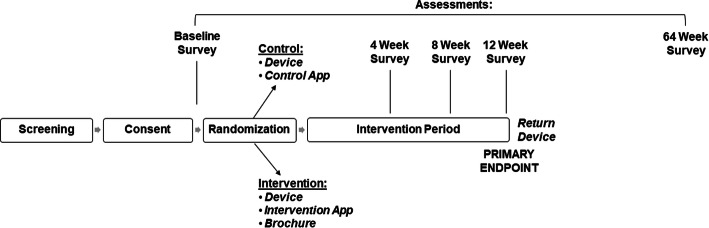
Study schematic

Following screening and consent, participants will complete the baseline survey and be randomized to condition. Participants will then be mailed their device and appropriate training materials and mobile app access within 1 week of randomization. Participants will be asked to wear the device and sync it with the mobile app every day for 12 weeks, regardless of planned physical or outdoor activities. Self-administered questionnaire data during the intervention period will be collected at week 4, week 8 and week 12 (end of intervention), and week 64 (1 year after the end of the intervention; the end of the following summer). Participants will be asked to return the device after their 12-week intervention period is complete (Fig. 1).

### Sample size {14}

The sample size was calculated to maximize power for the primary outcome (self-reported overall sun protection score) while also achieving moderate power for a key secondary outcome, experience of a sunburn during the intervention period. A sample size of 368 randomized (314 completed by assuming a 85% completion rate) will achieve 95% power to detect an effect size of 0.40 between intervention and control groups using a two-sided two-sample t-test assuming a significance level of 0.05. This effect size is similar to that found in an intervention to promote sun protection behaviors in high-risk individuals with the same primary outcome [14]. Importantly, it will also achieve 70% power to compare report of sunburn during the intervention period between the two groups (detect 20% of the control participants reporting a sunburn, as seen in our preliminary data [29], and 10% of the intervention participants reporting a sunburn) as statistically significant at 0.05 significance level. We assumed that 85% of those randomized would complete the 12-week follow-up survey, which resulted in a total planned sample size of 368 (184 per group).

### Recruitment {15}

HealthPartners Institute Center for Evaluation and Survey Research will recruit potential participants. Screening and recruitment will follow a two-step process. First, HealthPartners medical claims data will be queried to identify patients or members diagnosed with invasive cutaneous melanoma during the recruitment eligibility period (2010 to present). Recruitment will occur in the spring of each year with the study intervention running during the summer to account for seasonality. We will send potential participants a letter on HealthPartners letterhead introducing the general nature of the study and the elements of informed consent, and notifying the potential participant that a study interviewer will call them. The letter will state that the information obtained is confidential and that participation is voluntary. Next, within 2 weeks of sending each letter, an interviewer trained specifically on this study protocol will phone the potential participant to ask if they are willing to participate in the study, if they have not already opted out. For those interested in participating, the interviewer will obtain permission to share their contact information (name, address, phone number) with members of the University of Minnesota research staff. The University of Minnesota staff will then send an introductory email, which includes a link to complete online consent and HIPAA forms, and then leads directly to the online baseline survey to finalize the study registration process. In light of the COVID-19 pandemic, all study procedures were designed to be conducted remotely with no in-person contact with participants.

## Assignment of interventions: allocation

### Sequence generation {16a}

Following written consent and completion of the baseline survey, participants will be randomized with a 1:1 ratio to the intervention or control arm. Randomization will be stratified by age, gender, disease stage (I, II, III/IV), and years since melanoma diagnosis (< 2 years, 2–5 years, 6+ years). We will use block randomization to improve balance.

### Concealment mechanism {16b}

The randomization is implemented within REDCap.

### Implementation {16c}

The study statistician used R to generate random sequence numbers based on the stratified, block randomization scheme and uploaded the allocation list to REDCap. Following consent and screening, the study coordinator at the University of Minnesota will randomize participants within REDCap.

## Assignment of interventions: blinding

### Who will be blinded {17a}

The staff recruiting potential participants and the study investigators will be blinded to the randomization. Participants will be blinded in that they will not be informed of their randomized group or provided specific information about what to expect from the mobile app. The study coordinator will be aware of the randomized group during the study and the statistician will only be unblinded at the time of final analysis.

### Procedure for unblinding if needed {17b}

There are no plans to permit unblinding of participants.

## Data collection and management

### Plans for assessment and collection of outcomes {18a}

Study measures are collected via online surveys (in REDCap [15]) and the Shade device. The survey is accessible anywhere via an internet connection. Self-administered questionnaire data are collected at the start of the intervention (baseline; week 0), week 4, week 8 and week 12 (end of intervention), and week 64 (1 year after the end of the intervention; the end of the following summer). Survey measures are detailed in Table 1. Reliable and valid measures were included when available.

**Table 1:** Study measures

Measure	Purpose	Description	Weeks				
			0	4	8	12	64
Health behaviors [30]	Distraction	Measures of health behaviors, including alcohol, smoking, fruits, vegetables, vitamin D, height, weight	X	X	X	X	X
Physical activity [26]	Secondary outcome (unintended consequence)	Strenuous, moderate and mild activity times per week, and amount of time per session	X	X	X	X	X
Sun protection habits [20]	Primary outcome	Wearing a shirt with sleeves, wearing sunglasses, staying in the shade, using sunscreen, limiting time in the sun, and wearing a hat; Likert scale	X^a^	X^a^	X^a^	X^a^	X^a^
Sunburns [20]	Secondary outcome (efficacy)	Number of times had a red OR painful sunburn that lasted a day or more	X^b^	X^a^	X^a^	X^a^,^b^	X^b^
Sun exposure and other Protection measures [20]	Validation/comparison with device data	Exposure during weekday and weekend days, sunscreen details, indoor tanning	X^b^	X^a^	X^a^	X^a^	X^b^
Beliefs, intentions, self-efficacy regarding sun protection behaviors [31, 32]	Substudy (if intervention fails)	Reasoned action battery: intention, attitude, behavioral beliefs, perceived norms, normative beliefs, self-efficacy, efficacy beliefs	X			X	X
Sun protection knowledge	Secondary outcome (efficacy)	Knowledge of sun protection (items covered in brochure)	X			X	X
Hospital Anxiety and Depression Scale [27]	Secondary outcome (unintended consequence)	Screener for depression and anxiety used frequently in cancer survivors; Likert scale	X			X	X
Comorbidities [33]	Background	Presence of heart problems, hypertension, chronic back pain, arthritis, stroke, severe memory or concentration problems, lung diseases, stomach and/or intestinal problems, diabetes, depression, anxiety, neuropathy, and other cancer diagnoses	X			X	X
Diagnosis and treatment [33]	Background/potential moderators	Year diagnosis, location, stage, treatments received, recurrence, metastasis	X				
Fear of recurrence [34]	Background/potential moderator	Cancer and health worry	X			X	X
Demographics	Background/potential moderators	Sex, age, race/ethnicity, education, income, marital status, parent status, family history of cancer, skin phenotype	X				
Device use	Potential moderator	Self-reported and device-provided		X	X	X	
Device usability/satisfaction [28]	Secondary outcome	System Usability Scale				X	

^a^Time frame: past 4 weeks

^b^Time frame: past summer

Recruitment metrics will include the proportion of eligible individuals who agree to participate and the number of contacts required to recruit a participant. Measures of compliance and engagement will focus on device set-up and intervention usage, including time from recruitment to device use, average number of days the device is worn per week, and self-reported engagement/response to the UVR notifications on the mobile app. Study retention will be measured as the proportion of participants who complete the baseline and each of the follow-up surveys.

### Plans to promote participant retention and complete follow-up {18b}

Participants will be provided gift cards by mail throughout the study period as they complete study procedures to encourage survey completion. They will receive $20 following completion of the baseline survey, $10 for the week 4 survey, $10 for the week 8 survey, $60 for returning the device at the end of the study period and completing the week 12 survey, and $20 for the week 64 survey (for a total of up to $120).

### Data management {19}

Study staff will review all signed consent and HIPAA authorization forms for completeness at the time of participant entry into the study. Apart from the UVR and physical activity data that are automatically collected by the wearable devices, data collection will occur primarily using REDCap, which will be set up to ensure data are clean and ready for analysis. Reports within REDCap will be used to identify missing data.

### Confidentiality {27}

Participants will be asked to keep their mobile phone password protected to guard their confidentiality. All identifying participant information will be stored on a secure REDCap database. UVR data are stored in a de-identified fashion (study ID only) within the Shade database. Data transfer will only occur with de-identified data with encrypted transfer of all information containing protected health information between participants and study databases. Study data will be de-identified before data analysis. Only the researchers directly involved with the study will have access to the data. Identifying data will be stored until completion of the study and manuscript submission.

### Plans for collection, laboratory evaluation, and storage of biological specimens for genetic or molecular analysis in this trial/future use {33}

Not applicable.

## Statistical methods

### Statistical methods for primary and secondary outcomes {20a}

Statistical analysis will focus on evaluating the efficacy, unintended consequences, and implementation of the intervention in this RCT. The main analysis of the primary outcome (self-reported overall sun protection score) will be a two-sample two-sided *t* test comparing mean scores at the end of the intervention period (12 weeks) between intervention and control groups. Randomization should result in two groups that are similar on patient characteristics and other extraneous factors that may influence sun exposure and protection behaviors; however, we will compare the randomized groups by wave, gender, age, stage of disease, device use (low/high), and baseline measures of sun protection and exposure behaviors using two-sample *t* tests and chi-squared tests to identify potential confounding factors which may not be balanced between the two groups. We will supplement the primary analysis with a multivariate linear regression model to account for any identified group differences at baseline. A secondary analysis will explore changes in the sun protection score over the intervention period (baseline, weeks 4, 8, and 12) using a mixed effects regression model. This analysis will not be conducted until data collection in both waves has been completed.

Secondary analyses will focus on experience of sunburn, each self-reported sun protection behavior separately, intentional tanning, skin cancer knowledge, physical activity, and health behaviors by comparing the intervention and control participants over the study intervention period and 1-year post-completion. Analyses will be conducted using chi-squared and *t* tests as appropriate for univariate analyses at each time point and logistic and linear regression models for multivariate analyses. Daily UVR data (minutes of UVR exposure per day) will be summarized using the area under the curve (AUC) over the 12-week intervention period. Proper transformation (e.g., log-transformation) will be applied if the AUC data are skewed. The two groups’ AUC will be compared using similar methods as proposed above. The association between sun exposure and its potential moderating effect on sun protection behaviors during the intervention will be investigated using linear mixed effects models including main and interaction effects, adjusting for potential confounders. To address any potential intervention effects in the control group, we will compare the baseline and post-intervention sun protection behaviors scores using paired *t* tests, stratifying by randomization group. The proportions of survivors in the intervention and control groups with potentially clinically significant depression and anxiety will be compared using chi-squared tests and supplemented with multivariate logistic regression models. Finally, descriptive statistics will be used to summarize the implementation metrics both across and within the randomization group, both between Waves 1 and 2 and after completion of the study.

### Interim analyses {21b}

Not applicable.

### Methods for additional analyses (e.g., subgroup analyses) {20b}

We intend to complete pre-planned subgroup analyses by sex, age group, cancer stage, and time since diagnosis (stratifying factors). Subgroup analysis will allow us to evaluate the consistency of the effect of the invention versus control across important subgroups.

### Methods in analysis to handle protocol non-adherence and any statistical methods to handle missing data {20c}

We plan to perform all statistical analyses following intention-to-treat (ITT) procedures. That is, all subjects allocated to a treatment or intervention will be followed up, evaluated, and analyzed as members of that group or treatment arm regardless of their compliance with the assigned treatment. While it is not expected that participants will be incorrectly assigned, some may not use the device and certain features. For that reason, we will also summarize device use. A priori, we anticipate that the amount of use of the intervention will predict sun protection and exposure behaviors, though we will likely be underpowered for this analysis.

Every effort will be made to encourage participants’ compliance and data completeness. Nevertheless, we expect some amount of missing data. We plan to perform a comprehensive missing data analysis employing statistical methods that are valid under different missingness assumptions to determine if conclusions are sensitive to missing data. The primary missing data method will be multiple imputation using the Markov chain Monte Carlo (MI-MCMC) method [35, 36] assuming that data are missing at random—that is, the probability that an observation is missing can depend on the observed data but not on the missing data. We will include appropriate baseline variables into the MCMC procedure to make the conditional independent missing assumption less stringent. If the treatment group is associated with missingness, we will conduct multiple imputation for each treatment group separately [37]. The MI-MCMC method allows arbitrary missing patterns including intermittent missing and dropout. We will also perform a simple imputation analysis as a sensitivity analysis; specifically, the linear interpolation method will be used for intermittent missing (e.g., when people forget to charge or wear the device), while the last week average value carried forward method will be used for dropouts, assuming that the average of the last 7 days’ data will be a good estimate of the missing data.

### Plans to give access to the full protocol, participant level-data, and statistical code {31c}

Individual-level data collected in this study will not be released or shared with those outside of the research team. Access to the full protocol and statistical code can be requested from the principal investigator.

## Oversight and monitoring

### Composition of the coordinating center and trial steering committee {5d}

This study is a single-site study that will be coordinated by the study team (all members listed on this protocol) at the University of Minnesota, led by the PI. All staff participating in study activities are required to have CITI Human Subjects training and staff will be trained on each version of the protocol prior to implementation of that version (should modifications be required). The University of Minnesota Institutional Review Board will provide oversight and has approved this study. The HealthPartners Institutional Review Board also approved the protocol.

### Composition of the data monitoring committee, its role and reporting structure {21a}

This study carries minimal risk and therefore the principal investigator will assume responsibility for monitoring and reporting safety concerns/events to the University of Minnesota Institutional Review Board. The study coordinator, trained by the PI, will monitor participants’ device use daily along with survey completion. In addition, automatic alerts are generated within REDCap to notify the study coordinator of missing or incomplete surveys. The PI will also review for data completeness and monitor participant drop-out weekly.

### Adverse event reporting and harms {22}

The risks are minimal and no serious adverse events are expected; however, any adverse events will be collected both systematically and non-systematically. We do not anticipate adverse events with the exception of potential increases in depression and/or anxiety symptoms. Depression and anxiety measures will be included in surveys every 4 weeks during the intervention; these data will not be summarized until the time of trial completion. We also include qualitative fields in each survey to report additional concerns, along with providing contact information for the study coordinator. The principal investigator will report safety concerns/events to the University of Minnesota Institutional Review Board. Events requiring prompt reporting include any adverse event that requires a change to the protocol or consent form, any unauthorized disclosure of confidential information, any unresolved subject complaint, or any protocol deviation that results in harm or the unanticipated death of an enrolled subject. All adverse events will be reported in trial publications.

### Frequency and plans for auditing trial conduct {23}

The principal investigator and study coordinator will conduct ongoing auditing of study conduct. The Masonic Cancer Center Data and Safety Monitoring Council provides ongoing data and safety oversight for all investigator-initiated trials in the Masonic Cancer Center. The Data and Safety Monitoring Council reviews all interventional investigator-initiated clinical trials regardless of protocol type, e.g., therapeutic and supportive care at least annually from the time a protocol is opened to accrual until it is closed to accrual and all subjects have completed treatment. These trial progress reports cover trial activity at the Masonic Cancer Center and, if applicable, any affiliate site(s) and include assessment of expectancy, attribution, and seriousness of adverse events; monitoring findings; protocol deviations; dose-limiting toxicities and stopping rule events; and independent notification of safety concerns from the Institutional Review Board or PI. If the Data and Safety Monitoring Council identifies serious safety concerns, the Chair communicates these in writing to the trial PI with a specified timeframe for the PI to respond or resolve the issues, or requests a for cause audit to be conducted of the trial.

All active investigator-initiated trials may be subject to an internal audit by the Masonic Cancer Center of any aspect of trial conduct. Audits may include but are not limited to review of subject records, consent process and documentation, regulatory compliance, product accountability, PI oversight, and protocol adherence. In addition, the University of Minnesota Institutional Review Board requires annual progress reviews and conducts random audits of ongoing studies.

### Plans for communicating important protocol amendments to relevant parties (e.g., trial participants, ethical committees) {25}

All protocol modifications require review and approval from the University of Minnesota Institutional Review Board prior to implementation. As appropriate, trial participants will be informed of modifications and ClinicalTrials.gov will be updated accordingly.

### Dissemination plans {31a}

We propose a comprehensive communication strategy to disseminate findings to scientific audiences, dermatologists, health plans, and survivors. Upon publication of study results in scientific journals, we will work with the University of Minnesota’s communications staff to promote press coverage to expand their reach to the general population, as we have done with previous work. We will also present results at diverse scientific meetings including those of regional and national dermatological and oncological societies. If successful in improving sun behaviors in this population, we will approach participants from the study to serve as an advisory group, along with dermatologists and oncologists, to make recommendations on executing an effective communications plan and identify appropriate advocacy groups to inform of our results. Finally, we will present results to health plan systems to further the reach of the results.

## Discussion

Study recruitment officially began in May 2020; data collection of the primary outcome will be completed in Fall 2021, and we expect to report preliminary trial results in 2022. This study was ready to launch in March 2020, just prior to the issuance of “stay at home” orders in Minnesota due to the COVID-19 pandemic. While initially thinking to delay recruitment in Summer 2020, we ultimately decided to proceed with the study for a number of reasons. First, while we were originally concerned about the prospect of low sun exposure during the pandemic, our anecdotal observation was that in fact many individuals were getting as much, if not more, sun exposure than usual, as Minnesota’s policies did not prohibit outdoor activities. Second, we determined that with minor changes to our protocol and recruitment methods and by carrying out all study procedures remotely, the study was feasible to conduct even during a stay-at-home order. Third, we were concerned that Summer 2021 might be similar regarding the state of the pandemic, and therefore it was likely not worthwhile to delay the study or potentially lose the benefit of conducting two waves. Last, a considerable number of cancer and other health-related research studies have been halted or suspended, and therefore, conducting studies that can proceed safely allows for ongoing scientific progress. We are, however, pursuing a somewhat smaller number of participants in Wave 1 than originally planned (prior to the pandemic) to ensure feasibility of conducting the study while working from home, with reduced access to staff and other resources, and to gather preliminary data about recruiting during this time.

## Trial status

The protocol presented here is version 3.1 and was finalized on May 8, 2020. The first participant was formally consented on June 9, 2020 and recruitment is expected to be completed by July 30, 2021.

## Supplementary Information

**Additional file 1.**

## Abbreviations

AUC: Area under the curve
HIPAA: Health Insurance Portability and Accountability Act
ITT: Intention-to-treat
MCMC: Markov chain Monte Carlo
REDCap: Research Electronic Data Capture
RCT: Randomized controlled trial
UVR: Ultra-violet radiation
