# Supplementary material for: UVR-sensor wearable device intervention to improve sun behaviors and reduce sunburns in melanoma survivors: study protocol of a parallel-group randomized controlled trial
Source: Trials. 2020 Nov 23;21:959. doi: 10.1186/s13063-020-04881-3 (PMC7682122; doi:10.1186/s13063-020-04881-3)
Supplement: Supplementary file 1 — Additional file 1. [file 13063_2020_4881_MOESM1_ESM.pdf]

Consent Form

Title of Research Study: Wearable device intervention to improve sun behaviors in melanoma survivors

Investigator Team Contact Information: Rachel I. Vogel, Ph.D.
For questions about research appointments, the research study, research results, or other concerns, call the study team at:

Investigator	Study Staff
Investigator Name: Rachel I. Vogel<br>Investigator Departmental Affiliation: Department of Obstetrics, Gynecology and Women's Health<br>Phone Number: 612-624-6928<br>Email Address: isak0023@umn.edu	Study Staff: Katherine Brown<br>Phone Number: 612-624-9904<br>Email Address: meldstudy@umn.edu

Supported By: This research is supported by research grants from the American Cancer Society and Melanoma Research Alliance.

Key Information About This Research Study

The following is a short summary to help you decide whether or not to be a part of this research study. More detailed information is listed later on in this form.

What is research?

Doctors and investigators are committed to your care and safety. There are important differences between research and treatment plans:

- The goal of research is to learn new things in order to help groups of people in the future. Investigators learn things by following the same plan with a number of participants, so they do not usually make changes to the plan for individual research participants. You, as an individual, may or may not be helped by volunteering for a research study.
- The goal of clinical care is to help you get better or to improve your quality of life. Doctors can make changes to your clinical care plan as needed.

Why am I being asked to take part in this research study?

We are asking you to take part in this research study because you were diagnosed with invasive melanoma of the skin since 2010 and diagnosed and/or treated by physicians associated with HealthPartners.

What should I know about a research study?

- Someone will explain this research study to you.
- Whether or not you take part is up to you.
- You can choose not to take part.
- You can agree to take part and later change your mind.

Approved for use by UMN IRB
Effective on 4/1/2020
IRB Study Number: STUDY00006257

- Your decision will not be held against you.
- You can ask all the questions you want before you decide.

Why is this research being done?

The purpose of this study is to test a wearable technology device (like a FitBit) to monitor the sun exposure of individuals who have had a melanoma diagnosis. Previous research suggests that while some melanoma survivors change their health behaviors (physical activity, nutrition, sun exposure), many do not. Melanoma survivors, like other cancer survivors, are at risk of second cancer, making it critical that they make healthy choices.

How long will the research last?

We expect that you will be in this research study for approximately 3 months, with one additional follow-up survey 1 year later.

What will I need to do to participate?

You will be asked to complete online surveys at the beginning of the study and then at 4, 8, 12 and 64 weeks after starting the study. These surveys will ask questions related to your melanoma diagnosis, knowledge about skin cancer, health behaviors, and emotional health. You will then be asked to wear a device on your wrist, which we will provide, daily for 12 weeks during the summer.

More detailed information about the study procedures can be found under ***"What happens if I say yes, I want to be in this research?"***

Is there any way that being in this study could be bad for me?

You may not feel comfortable reviewing the information presented by the device or on the website. You are free to skip over any sections or not answer any question.

Will being in this study help me in any way?

We cannot promise any benefits to you or others from your taking part in this research. However, possible benefits include increased knowledge about melanoma and sun exposure habits.

What happens if I do not want to be in this research?

You do not have to participate in this research.

Detailed Information About This Research Study

The following is more detailed information about this study in addition to the information listed above.

How many people will be studied?

We expect about 370 people will participate in this study, all of whom live in Minnesota and neighboring areas.

What happens if I say "*Yes, I want to be in this research*"?

If you agree to be in this study, we would ask you to do the following things:

1. Complete a pre-study online survey. This survey includes questions related to your melanoma diagnosis, knowledge about skin cancer, health behaviors and emotional health.

Approved for use by UMN IRB
Effective on 4/1/2020
IRB Study Number: STUDY00006257

2. You will receive a wearable device in the mail that works with a mobile application (app). There are two versions of the mobile app and which one you get will be chosen by chance, like flipping a coin. Neither you nor the study staff will choose which one you receive. You will have an equal chance of being given either mobile app. You will not be told which version of the mobile app option you are getting, however the study staff will know.
3. Using the training materials provided, including written information regarding how to access the website and training materials demonstrating device set-up and use, you will be asked to set up the device and sync it with your mobile phone or device.
4. You will be asked to wear the device every day for 12 weeks during the summer, regardless of planned outdoor activities. You will also be asked to sync the device with your phone each day.
5. You will also receive messages on a weekly basis during the study reminding you to use the device.
6. You will be asked to complete follow-up online surveys at 4, 8, 12 and 64 weeks after starting the study. Data from the device (use and activity) will also automatically be provided to the study team once you update the mobile app with your device data.
7. Following the 12 week intervention, you will be asked to return the device.

What happens if I say "Yes", but I change my mind later?

You can leave the research study at any time.   You can leave the research study at any time and no one will be upset by your decision.

If you decide to leave the research study, contact the investigator (Rachel Vogel, email: isak0023@umn.edu, mailing address: 420 Delaware Street SE MMC 395, Minneapolis, MN 55455). Upon receipt of your request to discontinue participation in the study, the study team will stop contacting your regarding data collection and follow-up. Data collected prior to your withdrawal will be used in data analyses unless you request otherwise.

Choosing not to be in this study or to stop being in this study will not result in any penalty to you or loss of benefit to which you are entitled. This means that your choice not to be in this study will not negatively affect your right to any present or future medical care.

Will it cost me anything to participate in this research study?

Taking part in this research study will not lead to any costs to you.

What happens to the information collected for the research?

Efforts will be made to limit the use and disclosure of your personal information, including research study and medical records, to people who have a need to review this information. We cannot promise complete confidentiality. Organizations that may inspect and copy your information include the Institutional Review Board (IRB), the committee that provides ethical and regulatory oversight of research, and other representatives of this institution, including those that have responsibilities for monitoring or ensuring compliance.

Whom do I contact if I have questions, concerns or feedback about my experience?

This research has been reviewed and approved by an IRB within the Human Research Protections

Approved for use by UMN IRB
Effective on 4/1/2020
IRB Study Number: STUDY00006257

Program (HRPP). To share feedback privately with the HRPP about your research experience, call the Research Participants' Advocate Line at 612-625-1650 or go to https://research.umn.edu/units/hrpp/research-participants/questions-concerns. You are encouraged to contact the HRPP if:

- Your questions, concerns, or complaints are not being answered by the research team.
- You cannot reach the research team.
- You want to talk to someone besides the research team.
- You have questions about your rights as a research participant.
- You want to get information or provide input about this research.

You may also contact the HealthPartners IRB at 952-967-5025 if you have questions about this study.

Will I have a chance to provide feedback after the study is over?

The HRPP may ask you to complete a survey that asks about your experience as a research participant. You do not have to complete the survey if you do not want to. If you do choose to complete the survey, your responses will be anonymous.

If you are not asked to complete a survey, but you would like to share feedback, please contact the study team or the HRPP. See the "Investigator Contact Information" of this form for study team contact information and "Whom do I contact if I have questions, concerns or feedback about my experience?" of this form for HRPP contact information.

Will I be compensated for my participation?

If you agree to take part in this research study, you will be provided up to $120 in gift cards for your time and effort. You may also receive a $100 gift card during the study period if randomly chosen in the weekly lottery and wore/synced the device at least 5 days the previous week.

Use of Identifiable Health Information

We are committed to respect your privacy and to keep your personal information confidential. When choosing to take part in this study, you are giving us the permission to use your personal health information that includes health information in your medical records and information that can identify you. For example, personal health information may include your name, address, phone number or social security number. Those persons who get your health information may not be required by Federal privacy laws (such as the 1099 Rule) to protect it. Some of those persons may be able to share your information with others without your separate permission. Please read the HIPAA Authorization form that we have provided and discussed.

The results of this study may also be used for teaching, publications, or for presentation at scientific meetings.

Approved for use by UMN IRB
Effective on 4/1/2020
IRB Study Number: STUDY00006257

Consent Form

Your signature documents your permission to take part in this research. You will be provided a copy of this signed document.

____ __________________
Signature of Participant Date

____
Printed Name of Participant

____ __________________
Signature of Person Obtaining Consent Date

____
Printed Name of Person Obtaining Consent

Approved for use by UMN IRB
Effective on 4/1/2020
IRB Study Number: STUDY00006257
